# Ecophysiological and Anatomical Mechanisms behind the Nurse Effect: Which Are More Important? A Multivariate Approach for Cactus Seedlings

**DOI:** 10.1371/journal.pone.0081513

**Published:** 2013-11-28

**Authors:** Pablo Delgado-Sánchez, Laura Yáñez-Espinosa, Juan Francisco Jiménez-Bremont, Leonardo Chapa-Vargas, Joel Flores

**Affiliations:** 1 Facultad de Agronomía y Veterinaria, Universidad Autónoma de San Luis Potosí, Soledad de Graciano Sánchez, México; 2 Instituto de Investigación de Zonas Desérticas, Facultad de Ingeniería, Universidad Autónoma de San Luis Potosí, San Luis Potosí, México; 3 División de Biología Molecular, Instituto Potosino de Investigación Científica y Tecnológica, San Luis Potosí, México; 4 División de Ciencias Ambientales, Instituto Potosino de Investigación Científica y Tecnológica, San Luis Potosí, México; University of New South Wales, Australia

## Abstract

**Background:**

Cacti establish mostly occurs under the canopy of nurse plants which provide a less stressful micro-environment, although mechanisms underlying this process are unknown. The impact of the combination of light and watering treatments on *Opuntia streptacantha* (Cactaceae) seedlings was examined.

**Methods/Principal Findings:**

Ecophysiological [titratable acidity, osmotic potential (‘solute potential’, *Ψ_s_*), relative growth rate (RGR) and their components (NAR, SLA, and LWR)], anatomical (chloroplast density, chloroplast frequency, and cell area), and environmental [photosynthetic photon flux density (PPFD) and air temperature] sets of variables were analyzed, assessing relationships between them and measuring the intensity of the relationships. Three harvests were carried out at days 15, 30, and 45. *Ψ_s_* and acidity content were the most important responses for seedling establishment. The main anatomical and environmental variables were chloroplast density and water availability, respectively. *Opuntia streptacantha* seedlings establish better in the shade-watering treatment, due to higher *Ψ_s_* and acidity, unaffected chloroplasts, and lower PPFD. In addition, the chloroplasts of cells under high-light and non-watering treatment were clumped closer to the center of the cytosol than those under shade-drought, to avoid photoinhibition and/or to better distribute or utilize the penetrating light in the green plant tissue.

**Conclusions:**

*Opuntia* seedlings grow better under the shade, although they can tolerate drought in open spaces by increasing and moving chloroplasts and avoiding drastic decreases in their *Ψ_s_*. This tolerance could have important implications for predicting the impact of climate change on natural desert regeneration, as well as for planning reforestation-afforestation practices, and rural land uses.

## Introduction

Plant recruitment in arid environments often occurs only in years with above average rainfall or in safe sites under the canopy of nurse plants that provide shelter from high temperatures and low moisture. The nurse-protégé interaction is thought to be more frequent in harsh than in mild environments and appears to occur frequently in arid and semi-arid biomes [1]. The Cactaceae family includes the highest number of species protected by nurse plants [1]; for example, in the southern Chihuahuan Desert of Mexico, *Opuntia streptacantha* seedlings establish mostly under the canopy of nurse plants [2], [3]. However, physiological and anatomical mechanisms underlying the nurse effect on *Opuntia* seedlings are poorly known in arid environments [4] and the lack of this knowledge is impeding our understanding of these processes, e.g. there are no studies about the morphological [specific leaf area (SLA) and leaf weight ratio (LWR)] and physiological [net assimilation rate (NAR)] components of the relative growth rate (RGR), nor the anatomical responses such as chloroplast frequency, chloroplast density, and cell area.

Cactus plants generally exhibit CAM-photosynthesis, in which CO_2_ exchange occurs at night when the water vapor pressure difference between the air and the transpiring surfaces is lowest, resulting in high water-use efficiency in adult plants [5] but not necessarily during seedling stages and when water from rainfall is plentiful [6], [7]. The CAM pathway is very common in arid and semiarid environments, where plants are frequently exposed to low soil moisture and high solar radiation [6], [8]. There are numerous reports showing that incident photosynthetic photon flux density (PPFD) during daytime is positively correlated with nocturnal acid accumulation, in adult cacti [9], [10]. For cactus seedlings, Franco and Nobel (1989) [11] found that photosynthesis and growth of *Ferocactus acanthodes* under the canopy (diminished light) of nurse plants were lower than under direct sunlight. However, Gallardo-Vasquez and De la Barrera (2007) [12] found that nocturnal acid accumulation decreases for light - exposed cactus seedlings but increases for those shaded, regardless of their water treatment. Thus, the presence of CAM in shaded cactus seedlings can increase water-use efficiency and reduce the risk of photoinhibition [12]. It is possible that *Opuntia* seedlings established in open, highly illuminated spaces, show lower acid accumulation than those established in shade.

Diurnal movement of chloroplasts in the CAM plant *Zygocactus truncates* (epiphytic cactus) has also been reported under the combination of light and water stress [13], but not in *Opuntia* species or in cactus seedlings that require the protection of nurse plants. Chloroplasts are usually positioned on cell walls parallel to the leaf surface, but they can reorient along cell walls perpendicular to the leaf surface, parallel to the direction of incident light to avoid absorption [14]; [15].

Aridity is expected to increase in arid and semi-arid ecosystems due to global warming. Nobel (2010) [10] suggested that the future of succulents such as cacti is extremely bright under new climatic conditions expected in the 21^st^ century. Thus, understanding how water availability and light intensity influence the ecophysiological and anatomical performance of cactus seedlings is crucial in having a better understanding of the organism's dynamics, adaptation, and plasticity [16], as well as of the impact of climate change on natural desert regeneration.

In this paper, the effect of the combination of light and watering treatments (shade and watering, shade and non-watering, light and watering, and light and non-watering) has been examined on *O. streptacantha* seedlings. We hypothesized that shade of nurse plants and watering favor seedling development. *Opuntia streptacantha* Lem. (Prickly Pear) is a perennial arborescent cactus of economic interest which has edible fruit and young pads. It is distributed throughout the semiarid lands of central Mexico [17], [18]. In order to corroborate our hypothesis, we assessed three types of response variables: ecophysiological [titratable acidity, osmotic potential (‘solute potential’, *Ψ_s_*), and relative growth rate (RGR) and their components (NAR, SLA, and LWR)], anatomical (chloroplast frequency, chloroplast density, and cell area), and environmental (photosynthetic photon flux density (PPFD), air temperature, and soil water condition). In addition, we also analyzed the importance of ecophysiological and anatomical variables in order to know which is more important as mechanisms behind the nurse effect.

## Materials and Methods

### Study design

*Opuntia streptacantha* seeds were collected at Mexquitic de Carmona municipality of San Luis Potosi, Mexico (22°16′ N, 101°07′ W at 2,020 m asl), in the Southern Chihuahuan Desert, in 2007. The seed collection was carried out on a private land; the owner of the land gave permission to conduct the study on this site. This study did not involve endangered or protected species.

At May 15^th^, 2008, seeds were sown in plastic trays with Sunshine Mix # 3 substrate (Sun Gro Horticulture Canada Ltd), which were placed in a greenhouse and watered every other day. Four months later, on September 15^th^, 2008, the seedlings were transplanted into pots with soil from the *O. streptacantha* habitat and maintained at field capacity for 20 days, also in a greenhouse. All the experimental units were randomised within the glasshouse. Then, two treatments and two levels within each treatment were randomly assigned to 100 seedlings corresponding to a total of 25 replicates after treatment. Treatments included: neutral shade (25% light) with watering (SW) at field capacity every other day, neutral shade without watering (SNW), light (100% light) with watering (LW), and light without watering (LNW). The watering regime resembled natural conditions. The waterless treatments mimic dry periods with no rain. Treatments with water were intended to model years in which rains were frequent throughout the entire wet season. In addition, watering provided a good control of added water quantity to experimental units. Field capacity was determined in pots containing overwatered mixture and allowed to drain overnight. To reach field capacity, 200 mL water per pot was required. Treatments with high solar radiation simulated solar radiation in open spaces without vegetation, whereas shaded treatments represented the low solar radiation (≈75% of light reduction) found under shrubs in the area. All of these treatments allowed us to measure the ecophysiological and anatomical responses of seedlings to contrasting environmental conditions.

In order to simulate low solar radiation, plants were covered with a plastic film mesh which created shade; for high radiation treatments, plants were not covered. Seedlings in the low moisture treatments were not watered, while those in the high moisture treatments were watered every other day to field capacity during the entire course of the experiment.

The response variables were measured 15, 30, and 45 days after transplanting, and at these times three seedlings were harvested per treatment (light and watering) and the two levels within each treatment. At the same time, PPFD and temperature were measured using a portable fluorometer (pulse-amplitude modulated photosynthesis yield analyzer, Mini-PAM; Walz GmbH, Effeltrich, Germany). This instrument is equipped with a micro-quantum sensor to monitor PPFD and a thermocouple to measure temperature.

The following parameters were evaluated: osmotic potential (*Ψ_s_*), titratable acidity content, chloroplast density, chloroplast frequency, and cell area. In addition, chloroplast arrangement was described.

### Osmotic potential (*Ψ_s_*)

Osmotic potential was evaluated as an estimation of plant water status, because the accumulation of malate in the vacuole of CAM species causes a reduction in sap osmotic potential, which may favour water absorption. Water status measurements were completed at predawn. The experimental unit consisted of one seedling *per* pot to obtain sufficient sap extract. *Ex situ* determinations were made of the *Ψ_s_* of the sap obtained by mechanical compression [19]. *Ψ_s_* was recorded in C-52 sampling chambers connected to a Wescor HR-33T dew-point microvoltmeter (Wescor Inc., Logan, Utah, U.S.A.). Sampling chambers were calibrated prior to use with standard NaCl solutions [19].

### Titratable acidity content

Organic acid content, mainly malic acid, but also oxaloacetic acid and citric acid [6] in seedlings was estimated from titratable acidity, which was determined at 6:00 a.m., when its concentration is highest (6). Transverse samples of plant tissue were obtained from seedling shoots using a steel borer of cross section 0. 2 cm^2^. The plant material was sectioned and preserved in ethanol (80%) in 1.5 mL Eppendorf tubes. Titration was carried to neutrality with a 0.01 N NaOH solution to determine the organic acid concentration (mmol equiv. H^+^ m^−2^) *per* tissue according to Pearcy et al. (1991) [20].

### Photosynthetic area

To determine the photosynthetic area of *Opuntia streptacantha* stems, seedlings were scanned with an HP 2400 ScanJet scanner. The images were analyzed with the software ImageJ 1.40 g (Wayne Rasband National Institutes of Health, USA).

### Seedling growth

We measured relative growth rate (RGR) and its components at three time periods on harvested samples. The starting point for measuring changes in biomass was 15 days after transplanting. The calculations were realized using the formula Relative growth rate (RGR) =  Net assimilation rate (NAR, g cm^−2^ day^−1^) * Specific leaf area (SLA, cm^2^ g^−1^) * leaf weight ratio (LWR, g g^−l^), following Shipley (2000) [21]. NAR represents an increase in plant total biomass (TB) per total leaf (or photosynthetic) area (TLA) unit and time (T) unit (NAR = (TB_2_−TB_1_)/(T_2_−T_1_). 2/(TLA_1_ + TLA_2_), mg/day/cm^2^); NAR is a physiological component because it is a measure of whole-plant daily net photosynthetic rate weighted by the rate of change in plant carbon content [22]. SLA is a morphological component which is determined by leaf dry matter concentration and leaf thickness [23], [24], [25]. In a succulent cactus without leaves, such as *Opuntia*, the SLA is the specific photosynthetic structure area (SPSA =  photosynthetic structure area/photosynthetic structure dry mass at time t); LWR measures the allocation of biomass to leaves or photosynthetic organs *vs.* other plant parts (LWR =  leaf (photosynthetic structure) dry mass at time t/plant dry mass at time t) [24], [25].

### Light microscopy

Small samples of three *O. streptacantha* seedlings (five cells by seedling), including epidermis, hypodermis, and chlorenchyma were collected at 9:00 hours and immediately fixed with cold 4% glutaraldehyde in a phosphate buffer at pH 7.4, and dehydrated with graded ethanol series. Samples were hand-sectioned with a low profile microtome blade and the transverse sections were examined by light microscopy (Leica DM2000; Leica Microsystems Inc., Buffalo Grove, Il, USA) examination. Cell micrographs were obtained (LAS Imaging Software; Leica Microsystems Inc., Buffalo Grove, Il, USA) and digitized to determine the number of chloroplasts *per* cell and the cell cross-sectional area for every cell for five cells *per* micrograph (five micrographs *per* seedling *per* treatment). Chloroplasts were counted in one plane for each cell to estimate the number of chloroplasts on the top surface of each cell. Chloroplast density was calculated as the number of chloroplasts *per* 100 µm^2^ of cell area, and the chloroplast frequency as the number of occasions that chloroplasts are observed *per* grid unit within the cell, divided by the total grid number. Cell size was measured with the software ImageJ 1.40 g (Wayne Rasband National Institutes of Health, USA) using the microscope scale as reference.

### Statistical analysis

A canonical discriminant analysis was used to understand the complex relationship between three groups of original variables (ecophysiological, anatomical, and environmental) and the relative contribution of these variables (within each group) to explain the effect of our experimental design, describing the linear combination of the original variable coefficients (canonical variables) that maximally discriminates among groups.

Data were analyzed d using a canonical discriminant analysis that allowed the identification of differences among the combined treatments (SW, SNW, LW and LNW) mentioned previously, using data obtained from variables from individuals belonging to each group and by utilizing linear functions from the quantified variables. Then the groups of individuals were separated to maximize the variance among treatments and to minimize the variance within them. The standardized canonical coefficients show the contribution of each of the joint variables which were analyzed to each of the canonical functions, and individually indicate the relative importance of each variable [26], [27]. In addition, a classificatory discriminant analysis was used to estimate the number and percent of entities classified correctly or incorrectly into each group of combined treatments. Both analyses were carried out using PROC CANDISC and PROC DISCRIM procedures.

The relationship among the three sets of variables (ecophysiological, anatomical, and environmental) was determined by performing a generalized canonical correlation analysis [28]. Only selected variables (*P*<0.005) by the canonical discriminant analysis were included for canonical correlation analyses. From the generalized canonical correlation results, a simple canonical correlation analysis was applied to the original variables related to their canonical variates (ecophysiological, anatomical, and environmental), arranged as two sets of plant and environmental variables. The goal of canonical correlation is to analyze the relationships between two sets of variables, and to elucidate the relationship between the two sets of variables. One set of variables consists of response variables and the other set consists of explanatory variables. A redundancy analysis was also performed to calculate the variance in a set of original variables explained by a canonical variate of another set [26].

In addition, three-way ANOVA were performed for osmotic potential, titratable acidity, specific photosynthetic structure area, leaf weight ratio, and chloroplast density, with light, watering and time as main factors, in order to determine the differences among treatments in the variables that contributed significantly to the canonical functions. Tukey tests were carried out to discern specific differences among treatments. All statistical analyses were performed with SAS software (SAS Institute Inc.).

## Results

### Photosynthetic photon flux density (PPFD)

The PPFD is a measurement unit that expresses the light quantum in photons of solar energy (specific to wavelength) related specifically to photosynthesis. The daily total PPFD received by *O. streptacantha* seedlings was 1059±5.5 µmol m^−2^ s^−1^ for the light treatments and 267±4 µmol m^−2^ s^−1^ for the shade treatments. Similar photo flux densities were observed in the three harvests.

### Temperature

Temperatures for all treatments were of 39±0.55°C in the light treatments for the three harvests, and 33±0.38°C for the shade treatments.

### Canonical discriminant analysis

We determined that two discriminant functions accounted for 99% of the data set total variation, contributing significantly to the separation among treatments (Wilks' λ: *P*<0.0001, *n* = 36). The first function (eigenvalue of 17.69; *P*<0.0001) explained 93% of the total variation, and the second (eigenvalue of 1.17; *P*<0.0001) explained 6% of the total variation. The remaining function accounted for 1% of total variation and was interpreted as trivial.

**Figure 1:**
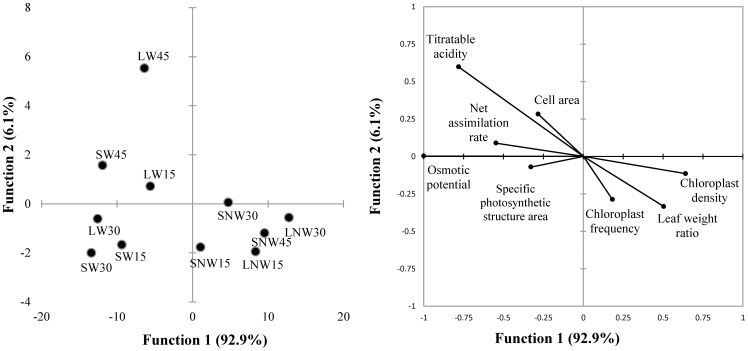
Correlations between the variables and their canonical variates (A); Scatterplot showing the canonical correlation between centroids of the first pair of canonical variates and lineal tendency line (B).

Variables that contributed significantly to the two canonical functions were present in the standardized canonical coefficients, and those that most contributed to centroid separation among treatments were *Ψ_s_*, chloroplast density, specific photosynthetic structure area, titratable acidity, and leaf weight ratio in the first function, as well as cell area and chloroplast frequency in the second function (Figure 1).

The classificatory discriminant analysis showed that centroids of each group were significantly different (*P*<0.0001). All of the observations were correctly classified for all treatments.

### Canonical correlation analysis

The generalized canonical correlation revealed that there was only one canonical variate. Vector defining canonical variates to produce the vector of the first canonical variates are shown in Table 1. The first eigenvalue (λ_1_ = 2.00) accounted for most of the variance of the first canonical variate, indicating that it is generated by only one common factor, and indicated that the three sets of variables are highly correlated and all had similar weight in determination of the first canonical variate. The second (λ_2_ = 0.68) and third eigenvalues (λ_3_ = 0.29) accounted for less of the variance. The first eigenvector elements (e_1_ = 0.63_ecophysiological_; 0.52_anatomical_; 0.56_environmental_) showed that the anatomical set had the lowest value. The results from the correlation matrix among canonical variates indicated that highest correlation between ecophysiological and environmental variates (0.65), followed by ecophysiological and anatomical (0.56), and the lowest between anatomical and environmental (0.19).

**Table 1:** Results from the first canonical variates vector.

Sets	Original variables	Canonical variates
Ecophysiology	Osmotic potential (*Ψ*_s_)	−0.808
	Titratable acidity	0.530
	Leaf weight ratio	−0.108
	Specific photosynthetic structure area	−0.230
Anatomy	Chloroplast density	1.00
Environment	PPFD	0.866
	Air temperature	−0.098

Table 2 shows correlations between the original variables and the canonical variates within sets. These correlation coefficients corroborated that *Ψ_s_* and titratable acidity were highly correlated with ecophysiological (their first canonical) variate, which means that these variables were important in the resolution of the canonical variate. The highly associated variable with anatomical variables was chloroplast density. With respect to the environmental variables, air temperature in the harvests and PPFD were highly correlated. These were the variables selected for the canonical correlation analysis.

**Table 2:** Correlation between original and canonical variables within groups.

Sets	Original variables	Canonical variates
Ecophysiology	Osmotic potential (*Ψ*_s_)	−0.808
	Titratable acidity	−0.304
	Leaf weight ratio	0.169
	Specific photosynthetic structure area	0.169
Anatomy	Chloroplast density	1.00
Environment	PPFD	0.866
	Air temperature	0.625

The first canonical correlation is 0.76 (84% of variance, *P*<0.004), however, the remaining correlation was not significant. Data for the first pair of canonical variates (cross-loadings) appear in Table 3. Percentage of variance (81%), redundancy (0.24), and canonical correlation (0.76) indicate that the first pair of canonical variates was highly correlated. The cross-loadings showed that *Ψ*_s_ was negatively correlated with PPFD, water treatment, titratable acidity, LWR, SPSA, and chloroplasts density. In addition, titratable acidity was correlated with chloroplast density, and both variables were correlated with PPFD and water treatment (Figure 1, Table 3).

**Table 3:** Canonical cross-loadings of the first pair of canonical variables.

		First Pair of Canonical Variates	
		Correlation	Coefficient
Mixed*	Osmotic potential (Ψ_s_)	−0.34	−1.59
	Titratable acidity	0.14	1.25
	Chloroplast density	0.18	−0.03
	Leaf weight ratio	−0.15	−0.27
	Specific photosynthetic structure area	−0.22	−0.01
Environmental	PPFD	0.59	0.74
	Air temperature	0.35	0.01

*Anatomical and ecophysiological variables.

Only selected variables (P<0.005) by the canonical discriminant analysis were included for canonical correlation analyses.

### ANOVA results

We determined the differences among treatments in the variables that contributed significantly to the canonical functions: osmotic potential, titratable acidity, specific photosynthetic structure area, leaf weight ratio, and chloroplast density.

**Figure 2:**
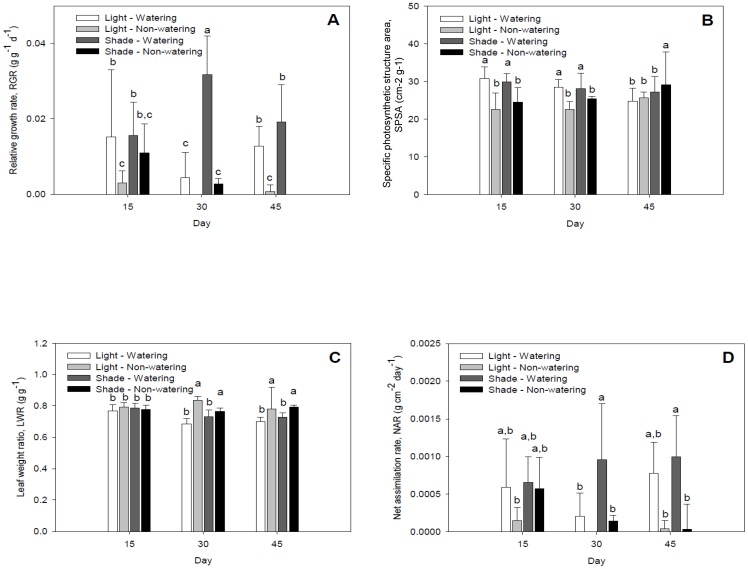
Responses of *Opuntia streptacantha* seedlings under combined water and light treatments at 15, 30 and 45 days. a) osmotic potential (MPa) and, b) titratable acidity (mmol equiv. H^+^ m^−2^). Bars represent the means ±SE (*P*<0.001; n = 3). Different letters indicated differences between treatments and days.

The osmotic potential was more affected under drought and high-light intensity than under drought and low-light intensity at the three times measured (Figure 2). Tritatable acidity was lowest under drought and high-light intensity at days 15 and 30, but at day 45 the acidity content was lowest under both drought and high- and low-light intensity (Figure 2).

**Figure 3:**
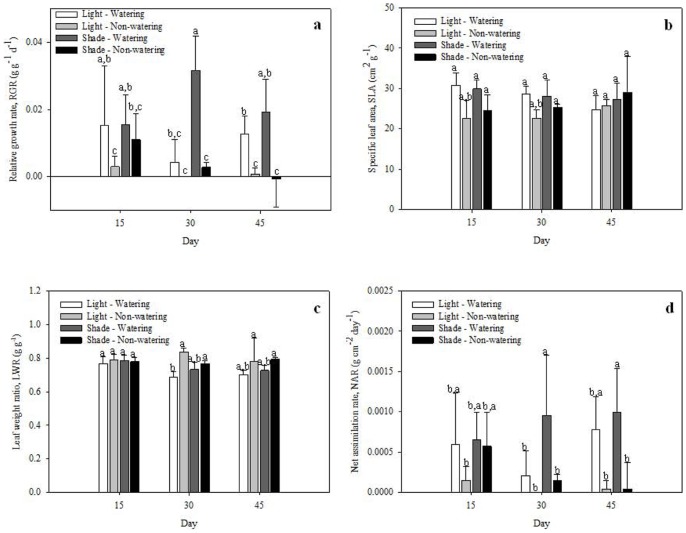
Responses of *Opuntia streptacantha* seedlings under combined water and light treatments at 15, 30 and 45 days. a) Relative growth rate (RGR; g g^−l^ day^−1^); b) specific photosynthetic structure area (SPSA; cm^2^ g^−1^); c) leaf weight ratio (LWR; g g^−l^), and d) net assimilation rate (NAR; g cm^−2^ day^−1^). Bars represent the means ±SE (*P*<0.001; n = 3).

RGR and NAR were not important to the canonical functions, but they also were affected by drought, high solar radiation, and time (*P*<0.001; Figure 3). The lowest RGR was found under combined drought and both high- and low- solar radiation treatments at days 15, 30 and 45. This also provided the highest RGR under combined watering and low solar radiation treatments at days 15, 30 and 45, as well as under watering and high solar radiation at day 15. The highest NAR was found under watering and low-light intensity at days 30 and 45.

The highest specific photosynthetic structure area was found under combined watering and both high- and low- solar radiation treatments at days 15 and 30, as well as under low-light intensity and drought at day 45 (*P*<0.001; Figure 3). The highest leaf weight ratio was found under combined drought and both high- and low- solar radiation at days 30 and 45 (*P*<0.001; Figure 3).

**Figure 4:**
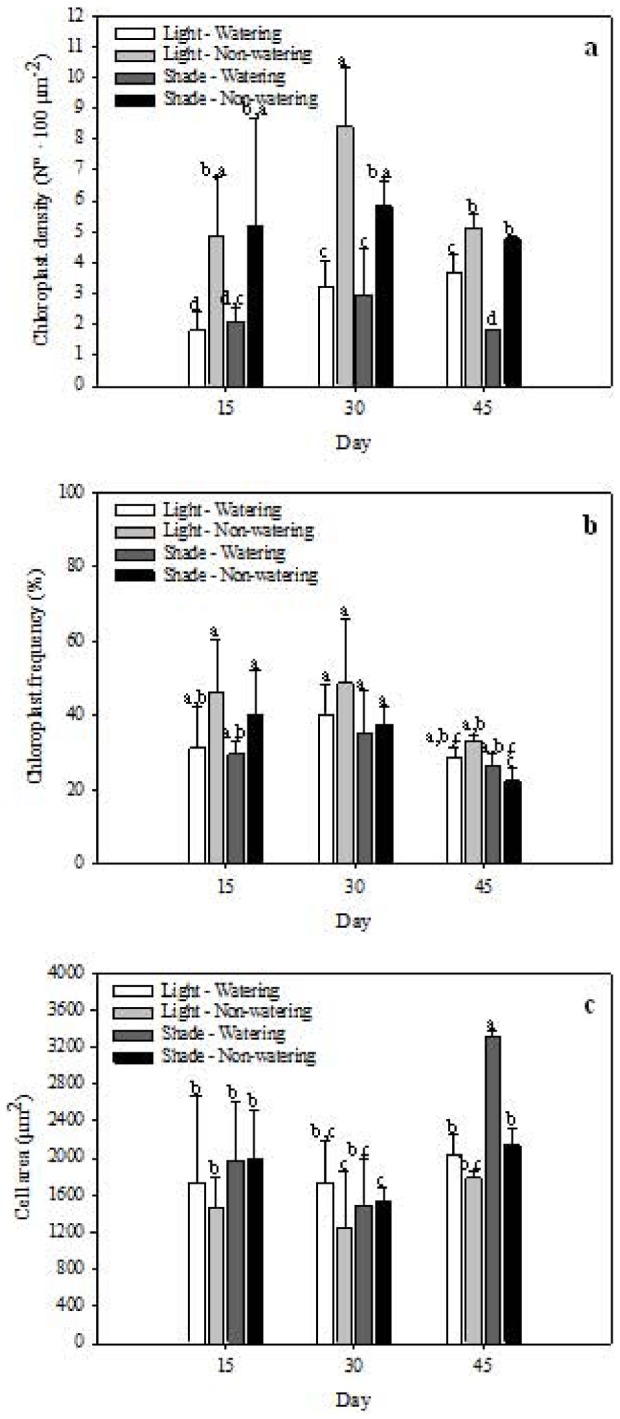
Responses of *Opuntia streptacantha* seedlings under combined water and light treatments at 15, 30 and 45 days. a) chloroplast density (N° 100 µm^−2^); b) chloroplast frequency (%) and, c) cell area (µm^2^). Bars represent the means ±SE (*P*<0.001; n = 3).

Chloroplast density was higher under non-watering (both shade and light) than under watering treatments across measured times, but it was highest under combined light and non-watering treatment at day 30 (*P*<0.001; Figure 4).

Chloroplast frequency and cell area were not important to the canonical functions, the former was lightly affected by the treatments because it was lower under watering (both high- and low- light intensity) at day 15, as well as under all combined treatments at day 45 (*P*<0.001; Figure 4). The highest cell area was found at shade-watering treatment after 45 days of the experiment (*P*<0.001; Figure 4).

### Chloroplasts arrangement

**Figure 5:**
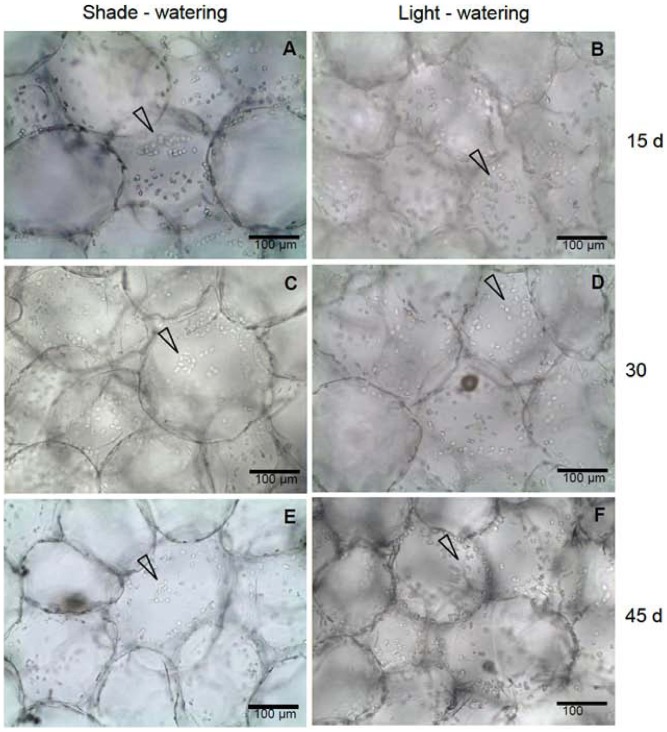
Chloroplasts arrangement along cell walls of *Opuntia streptacantha* seedlings under shade – watering (SW) and light – watering (LW) treatments. SW (a, c, e) and LW (b, d, f) at 15, 30 and 45 days, respectively. An arrow indicates the chloroplasts arrangement.

**Figure 6:**
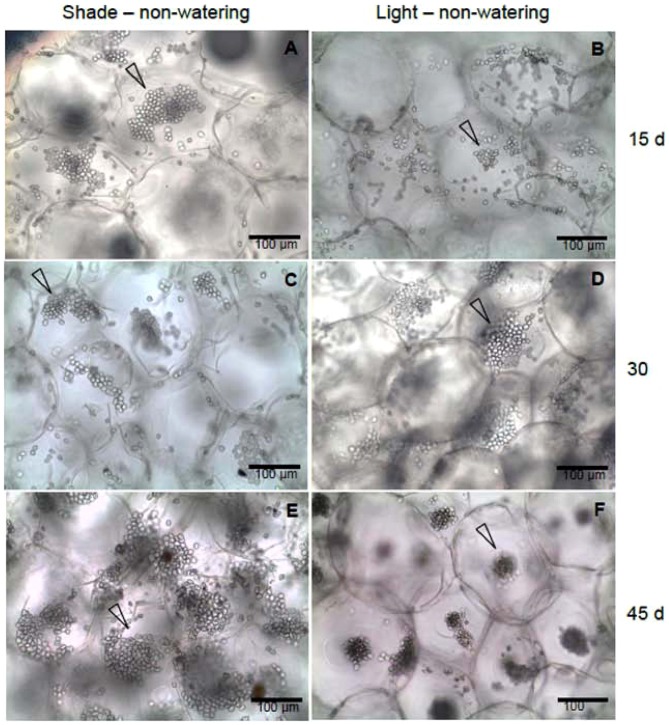
Chloroplasts arrangement of *Opuntia streptacantha* seedlings under shade – non-watering (SNW) and light – non-watering (LNW) treatments. a) SNW at 15 days, chloroplasts clumped in one area of the cytosol (arrow); b) LNW at 15 days, chloroplasts clumped in the peripheral cytosol (arrow); c) SNW at 30 days, chloroplasts clumped in one area of the cytosol (arrow); d) LNW at 30 days, chloroplast clumped located close together at adjoining cells (arrow); e) SNW at 45 days, chloroplasts clumped in one area of the cytosol (arrow) and f) LNW at 45 days, chloroplasts densely clumped in the center of the cytosol (arrow).

We found different chloroplast arrangements among the treatments in chlorenchyma cells. For cells from the watering treatment groups in both light and shade conditions, chloroplasts were always dispersed in the cytosol of the cells in the three harvests (Fig. 5a–f). The chloroplasts of cells from the LNW treatment group show a gradual aggregation toward the center of the cytosol for the three harvests, but this effect is most pronounced for the last harvest (Fig. 6b, d, f). A similar response of chloroplast in cells from the SNW group is observed, but chloroplasts are less aggregated than in cells of the LNW treatment group (Fig. 6a, c, e). In this sense, a similar behavior of chloroplast arrangement is observed when the seedlings were exposed to non-watering treatments, particularly at 45 days of the SNW treatment in comparison to LNW treatment at 30 days.

## Discussion

### Ecophysiological and anatomical responses: Which is more important as mechanism behind the nurse effect?

In this study, ecophysiological responses are the most important for *O. streptacantha* seedling growth, followed by anatomical responses. In terms of seedling biology, ecophysiological variables such as *Ψ_s_* and titratable acidity are very important for CAM plants, because acidity is regulated mainly by the water status of the plant [5], [8], [29], [30], [31], [32], [33], [34]. In this case, we found that *Ψ*_s_ of *O. streptacantha* seedlings is negatively correlated with titratable acidity. Interestingly, the *Ψs* was not particularly low (−1.5 MPa) for seedlings in the most stressful treatment (light and non-watering) at 45 days, which is similar to findings for adult *Opuntia* plants, *i.e.* during periods of high water stress, stem tissue water potentials are characteristically −1.2 to −1.6 MPa [32]. Our results show that *Opuntia* seedlings have the ability to osmotically adjust to tolerate drought, similar to findings for adult *Opuntia* plants, in which acidity content and *Ψ_s_* decrease in cladodes under drought conditions [32], [35]. The importance of water status for the growth of *O. streptacantha* seedlings is also in agreement with studies on other cactus seedlings [12], [36].

### Main ecophysiological mechanisms behind the nurse effect

Shade and higher soil humidity under nurse plants are two very important environmental factors facilitating seedling establishment [1]. Our results clearly show that for seedlings under shade the water status (in this case, the osmotic potential) and the acidity content did not decrease as rapidly as in high light conditions; this protection under nurse plants slows the effects of stress. Thus, *Opuntia* seedlings appear to have adapted to the protection of the shade of nurse plants, similar to findings for other cacti [12].

Growth frequently decreases under low water availability in a large number of species [37], [38], [39], including globose cactus seedlings [4]. However, RGR of *O. streptacantha* seedlings was not important inside the ecophysiological variables, probably because our study species is an obligatory CAM-photosynthesis [40] and other cactus seedlings could be CAM facultative [41], [42]. Similar findings were found under drought for *Clusia hilariana* seedlings, another obligatory CAM-photosynthesis plant [43]. Nevertheless, we found differences among treatments for RGR, having higher RGR in watering treatment. Interestingly, we also found higher cell size in the same treatment.

### Main anatomical mechanisms behind the nurse effect

The chloroplasts in cells of many plants can be displaced and rearranged in accordance with the incidence of PPFD, with chloroplasts exhibiting maximum surface area at low PPFD and aggregated under high PPFDs [44]. Light-induced chloroplast movement is an adaptive response [45], because the aggregation of chloroplasts tends to avoid photoinhibition and/or to better distribute or utilize the penetrating light in the plant leaf tissue [46], [47], [48]. We suggest that *Opuntia* seedlings established in open, highly illuminated spaces show chloroplasts movement in order to tolerate light and/or water stress. In this study, we found that chloroplasts are highly clumped in the center of the cell under high solar radiation and drought, which is in agreement with findings that chloroplast aggregation occurs under combined light and water stress [13], [49]. This behavior is also observed under shade and drought treatment, although to a lesser degree, chloroplasts also aggregate. Indeed, chloroplast arrangement is similar for LNW treatment at day 30 and SNW treatment at day 45.

Under field conditions, *O. streptacantha* seedlings are primarily found in shade under nurse plants [2], [3]. Hence, we suggest that the chloroplasts of seedlings under nurse plants move to maximize photosynthesis, because light coming through the canopy provided by nurse plants is weak, diffuse, and transient owing to the movement of the sun, as proposed by Park et al. (1996) [50] for shade plants. Interestingly, seedlings under non-watering treatments, both in light and shade, showed higher chloroplast density, contrary to that described for other plants in which the number of chloroplasts decreases under water stress or when exposed to intense light [51], [52], [53]. This could be a mechanism used by *O. streptacantha* seedlings to maintain photosynthetic activity under stress conditions.

Under drought condition with lower *Ψ_s_*, chloroplast clumping was also correlated with high chloroplast density and titratable acidity. Chloroplasts move towards a position of higher CO_2_ concentration for efficient photosynthesis [54], because higher concentration of malate in CAM plants is found in the vacuole. Thus, chloroplasts are closer to the vacuole for the transport of malate.

## Conclusions

There are clearly complex interactions between drought and light that promote different ecophysiological and anatomical adaptations, which have direct consequences in the seedling growth under nurse plants or in open spaces. Ecophysiological variables are the most important for *O. streptacantha* seedlings, followed by anatomical and environmental variables. Ecophysiological responses are more sensitive to environmental conditions, and these would be mainly affecting anatomical responses, but at the same time, anatomical variables are affected in less degree by environmental conditions than ecophysiological variables. Water stress decreased the osmotic potential under drought and high-light intensity at the three measured times. Tritatable acidity was lowest under drought and high-light intensity at days 15 and 30, but at day 45 the acidity content was lowest under both drought and high- and low-light intensity. The lowest RGR was found under combined drought and both high- and low- solar radiation treatments at days 15, 30 and 45. The highest NAR was found under watering and low-light intensity at days 30 and 45. The highest specific photosynthetic structure area was found under combined watering and both high- and low- solar radiation treatments at days 15 and 30, as well as under low-light intensity and drought at day 45. The highest leaf weight ratio was found under combined drought and both hig- and low- solar radiation at days 30 and 45. Chloroplast density was higher under non-watering (both shade and light) than under watering treatments at days 15, 30 and 45, but it was highest under combined light and non-watering treatment at day 30. Chloroplast frequency was lower under watering (both high- and low- light intensity) at day 15, as well as under all combined treatments at day 45. The highest cell area was found at shade-watering treatment after 45 days.

We found that *O. streptacantha* seedlings grow well in exposed sites with watering, but these conditions are not so common in arid and semi-arid environments. Thus, these plants can tolerate high-light and drought in open spaces by increasing and moving chloroplasts and also avoiding drastic decrease in their osmotic potential, although they could suffer damage in longer water stress periods. Higher soil humidity under nurse plants could be longer during the year than in open sites, for this reason, *O. streptacantha* seedlings establish better in conditions simulating microhabitat under the shade of nurse plants with high soil humidity in the rainfall period, having higher *Ψ_s_* and titratable acidity, as well as dispersed (unaffected) chloroplasts. These results could have important implications for predicting the impact of climate change on natural desert regeneration, as well as for planning reforestation-afforestation practices, and rural land uses.
