# Correction: Ecophysiological and Anatomical Mechanisms behind the Nurse Effect: Which Are More Important? A Multivariate Approach for Cactus Seedlings

**DOI:** 10.1371/journal.pone.0095405

**Published:** 2014-04-10

**Authors:** The PLOS ONE Staff

Figure 2 is incorrect. The authors have provided a corrected version here.

**Figure 2:**
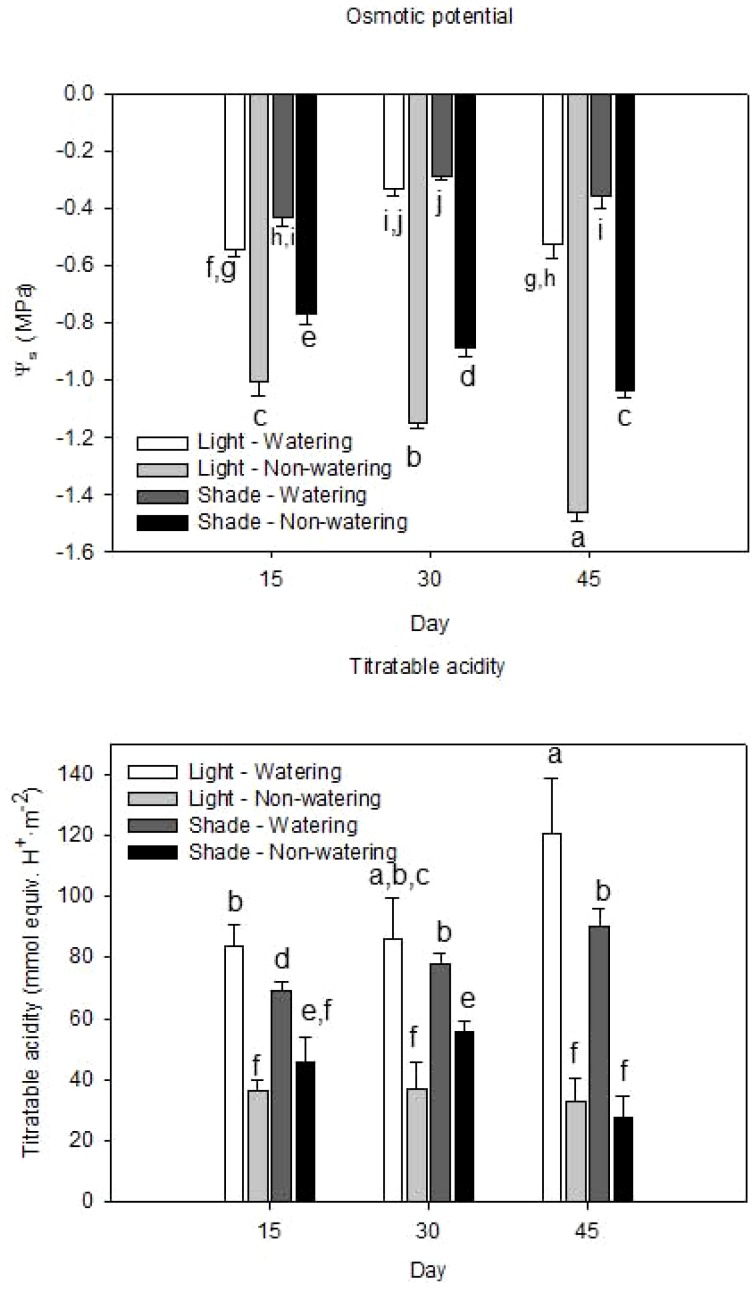
Responses of *Opuntia streptacantha* seedlings under combined water and light treatments at 15, 30 and 45 days. a) osmotic potential (MPa) and, b) titratable acidity (mmol equiv. H+ m-2). Bars represent the means ± SE (P < 0.001; n  =  3). Different letters indicated differences between treatments and days.
